# Evaluation of imputation accuracy using the combination of two high-density panels in Nelore beef cattle

**DOI:** 10.1038/s41598-019-54382-w

**Published:** 2019-11-29

**Authors:** Priscila Arrigucci Bernardes, Guilherme Batista do Nascimento, Rodrigo Pelicioni Savegnago, Marcos Eli Buzanskas, Rafael Nakamura Watanabe, Luciana Correia de Almeida Regitano, Luiz Lehmann Coutinho, Cedric Gondro, Danísio Prado Munari

**Affiliations:** 1 Departamento de Ciências Exatas, Faculdade de Ciências Agrárias e Veterinárias, Universidade Estadual Paulista, Jaboticabal, São Paulo Brazil; 2 Centro Universitário de Adamantina, Adamantina, São Paulo Brazil; 3 Instituto de Zootecnia, Sertãozinho, São Paulo Brazil; 4 Centro de Ciências Agrárias, Universidade Federal da Paraiba, Areia, PB Brazil; 5 Embrapa Pecuária Sudeste, São Carlos, São Paulo Brazil; 6 Departamento de Zootecnia, Escola Superior de Agricultura Luiz de Queiroz, Universidade de São Paulo, Piracicaba, São Paulo Brazil; 7 Department of Animal Science, College of Agriculture & Natural Resources, Michigan State University, East Lansing, Michigan USA

**Keywords:** Animal breeding, Genetic markers

## Abstract

This study compared imputation from lower-density commercial and customized panels to high-density panels and a combined panel (Illumina and Affymetrix) in Nelore beef cattle. Additionally, linkage disequilibrium and haplotype block conformation were estimated in individual high-density panels and compared with corresponding values in the combined panel after imputation. Overall, 814 animals were genotyped using BovineHD BeadChip (IllumHD), and 93 of these animals were also genotyped using the Axion Genome-Wide BOS 1 Array Plate (AffyHD). In general, customization considering linkage disequilibrium and minor allele frequency had the highest accuracies. The IllumHD panel had higher values of linkage disequilibrium for short distances between SNPs than AffyHD and the combined panel. The combined panel had an increased number of small haplotype blocks. The use of a combined panel is recommended due to its increased density and number of haplotype blocks, which in turn increase the probability of a marker being close to a quantitative trait locus of interest. Considering common SNPs between IllumHD and AffyHD for the customization of a low-density panel increases the imputation accuracy for IllumHD, AffyHD and the combined panel.

## Introduction

Genomic selection is now widely used in dairy cattle, but the beef industry is still trailing behind in the adoption of this technique due to the large number of breeds being used around the world and the challenge of building up a large enough reference population for each breed that will enable industry-relevant levels of accuracy for genomic breeding values^1^. Arguably, the best way to increase prediction accuracy is by increasing the number of animals with varying genotypes and phenotypes, although this comes at a high cost. Among other factors, the number of markers used for prediction can influence accuracy^2^ since the use of a large number of markers increases the probability of finding markers in high linkage disequilibrium (LD) with the quantitative trait loci (QTL).

There are two commercial high-density panels available for bovine species^3^: the BovineHD BeadChip (Illumina) and the Axion Genome-Wide BOS 1 Array Plate (Affymetrix). The Illumina panel contains approximately 777,000 single-nucleotide polymorphisms (SNPs) distributed homogeneously across the genome, whereas the Affymetrix panel contains approximately 640,000 SNPs, selected to reduce possible redundancy in the coverage of SNPs that are in high LD. Prices have come down since these chips were released, but they are still more expensive than the various lower-density panels (in the range of 20k–80k SNPs). For this reason, the bulk of genotyping is still performed using low- or medium-density panels. Additionally, important animals were previously genotyped with different panels and need to be used for studies or prediction, but it is difficult in terms of cost to regenotype these animals, or the DNA is no longer available.

A solution to combine these various panels is to impute genotypes from low-density panels to high-density panels and then use the imputed data for genomic prediction^4,5^. Many studies have investigated imputation from low- to high-density panels using the Illumina platform^6–9^. However, imputation studies with the high-density Affymetrix bovine panel are more limited^10,11^. High imputation accuracy is important for genomic prediction and genome-wide association studies^12^, as it has been shown that low imputation accuracy can affect prediction accuracies and the resolution of QTL regions in association studies^13,14^. Moreover, imputation from low- to high-density panels has been shown to be an efficient method to reduce the costs of genomic selection while still capturing most of the accuracy advantages of the high-density panels^15,16^. Additionally, imputation can be used to combine genotyped data from different chips^17^, resulting in higher-density panels and stronger LD with QTL. This improves the identification of haplotype blocks and the accuracy of the results from methodologies that use LD - and it is a critical step for industry to homogenize heterogeneous datasets.

The genomic information used in analyses could be enriched by using different panels of SNPs; however, combining the different panels that are present may be a challenge. Thus, recommendations on how to best combine different panels are required to take advantage of SNP information without increasing the cost. How well genotypes are imputed depends on the genetic relationship, reference population size, panel density, LD and minor allele frequency^18,19^. Therefore, a low-density panel that can be imputed with high accuracy to both high-density panels available for bovine species would be most useful for genomic prediction.

The first aim of this study was to compare imputation accuracies for Nelore beef cattle using two lower-density commercial arrays and a set of customized panels that we designed. Imputation accuracies were evaluated for each of the two bovine high-density panels and then for a pooled panel combining all SNPs from the two arrays. The second aim was to estimate the LD and haplotype block conformation using the high-density panels before and after imputation.

## Methods

### Ethical statement

All experimental procedures involving steers in this study were performed in accordance with the relevant guidelines (Protocol CEUA 01/2013) as approved by the Institutional Animal Care and Use Committee (IACUC) of the Brazilian Agricultural Research Corporation (EMBRAPA) and sanctioned by the president Dr. Rui Machado.

### Data description and quality control

Data from 34 Nelore bulls and 780 male offspring born in 2007, 2008, and 2009 were provided by Embrapa Pecuária Sudeste. The animals were maintained on farms located in São Carlos (Embrapa Pecuária Sudeste), in Campo Grande (Embrapa Gado de Corte), and on private farms in Mato Grosso and Mato Grosso do Sul in Brazil. The bulls were chosen to be representative of the main lines and genealogies of Brazilian Nelore and to minimize the kinship among them. Overall, all 814 animals were genotyped with the BovineHD BeadChip (Illumina), and 93 animals (23 bulls and 70 offspring) were also genotyped using the Axiom Genome-Wide BOS 1 Array Plate (Affymetrix). The genomic structure of this population was studied by Mudadu *et al*.^20^.

For quality control, SNPs were excluded if they were located in non-autosomal regions with an unknown position, had a Hardy-Weinberg equilibrium p-value of less than 10^−5^ or had an average call rate of less than 0.98. Animals with a call rate of less than 0.90 were also excluded. After quality control, 809 animals with 509,107 SNPs remained in the high-density Illumina (IllumHD) panel, and 93 animals with 427,875 SNPs remained in the high-density Affymetrix panel (AffyHD).

### Panels used for imputation

A combined panel (CP) was then generated by pooling the SNPs from IllumHD and AffyHD. The overlap between the two panels was low, with only 56,646 SNPs in common after quality control. Differences in SNP calls for SNPs that were common to the two panels were solved considering the call from the IllumHD panel due to the higher call rate for the majority of SNPs in this panel. The CP consisted of 880,336 SNPs.

To evaluate the imputation accuracy of low-density panels versus high-density panels (IllumHD, AffyHD, and CP), SNPs from the genotypes of IllumHD were masked to mimic the GeneSeek Genomic Profiler LD v2 array (20 kCom) and the Illumina BovineSNP50 v2 BeadChip (50 kCom). The 20 kCom and 50 kCom panels contained 15,575 and 27,946 SNPs, respectively.

**Figure 1:**
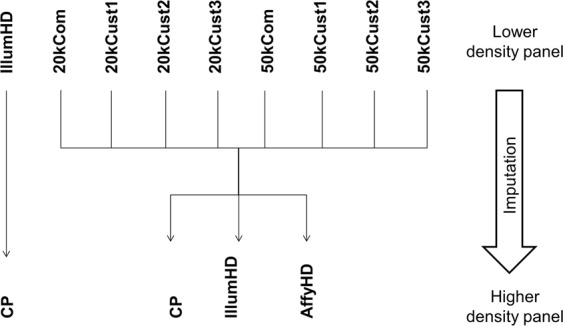
Organogram from the imputations studied in the present study. IllumHD - BovineHD BeadChip panel; 20 kCom - a panel reduced to GeneSeek Genomic Profiler LD v2; 50 kCom - a panel reduced to BovineSNP50 v2 BeadChip; Cust1, Cust2 and Cust3 - six custom panels with densities of 20k and 50k; AffyHD - Axion Genome-Wide BOS 1 Array Plate panel; and CP - combined panel formed using the IllumHD and AffyHD panels.

Three different methods were used to customize panels using the subset of SNPs common to both IllumHD and AffyHD (56,646 SNPs). In all methods, we selected the same number of SNPs as in the two commercial panels above (20 kCom and 50 kCom). For the first method (20kCust1 and 50kCust1) we selected SNPs that had the highest LD with SNPs from the commercial panels (20 kCom and 50 kCom). The second method (20kCust2 and 50kCust2) also took LD into account but additionally discarded SNPs with a minor allele frequency (MAF) less than 0.09 in either of the two high-density arrays. With this MAF value (0.09), all the customized panels have the same number of SNPs; use of other MAF cut-off values would result in a different number of SNPs for the second customized panel, which would not allow fair comparison with the other panels. For the third method, SNPs were split into windows of three SNPs each, then the MAF of each SNP was multiplied by the LD between that SNP and the other two in the window. Finally, for each window, the SNP that had the highest value for the sum of the results was selected to obtain 20 kCust3. A window containing two SNPs each was used for the 50 kCust3 panel. The LD was calculated as r^2^ in accordance with Hill and Robertson^21^. The imputation studies are shown in Fig. 1.

### Imputation

We used FImpute v.2.2b^17^ for the imputation analysis because it accounts for population and kinship information. In general, the oldest animals were genotyped using the Affymetrix high-density panel; therefore, the conditions used for all imputations included the 23 bulls in the reference population, and the other genotypes (786 animals) were used as the population to be imputed.

The IllumHD panel was imputed to the CP panel. All lower-density panels (2 commercial and 6 custom panels) were imputed up to IllumHD, AffyHD and CP. In the case of IllumHD, the imputation accuracy for each of the 786 animals was estimated, and among these, 273 were not offspring of the sires in the reference population. The imputation accuracies for AffyHD and CP were estimated only for the animals that had genotypes for AffyHD (70 offspring). Of these, 16 had no sire in the reference population. Due to the low number of animals from which the average imputation accuracies were estimated for these panels, imputation accuracies from lower-density to IllumHD panels were also estimated using the same 70 animals, and averages were compared with those obtained considering all 786 animals.

The accuracies were estimated as the error obtained from comparing the imputed marker alleles with the observed alleles and then calculating the proportion of correctly imputed genotypes (PROP). The allelic correlation (COR) between the imputed and observed genotypes was also calculated. The accuracies measured by PROP and COR were calculated in two ways, wherein for imputation comparison among the panels these measures were calculated by animal and with the purpose of verifying the accuracies along chromosomes and evaluating the relationship between accuracy and MAF, the PROP and COR were calculated by SNP. The relationship between the two accuracy measures and MAF was examined, establishing ten groups of SNPs using a MAF interval of 0.05. The accuracies observed across different chromosomes were evaluated for all imputed datasets.

### Linkage disequilibrium and haplotype blocks

Two LD measures for all panels were estimated using the correlation coefficient between alleles from two loci (r^2^)^21^ and |D′|^22^. Both measures were calculated using PLINK^23^. The LD between all pairs of SNPs was calculated within a 500-kb window, and the rate of decay was determined by calculating LD means for each 5 kb of distance between the SNPs, resulting in 100 bins. When the LD was calculated in the CP, pairs of alleles already observed in the IllumHD and AffyHD were found. The LD between pairs of alleles that were absent from the individual high-density panels (New_LD) was also calculated.

Additionally, the r^2^ means were calculated between SNPs that were present in lower-density panels and SNPs that were present only in higher-density panels. This mean was calculated for pairs of SNPs with a distance equal to or lower than the average distance between pairs of contiguous SNPs in the lower-density panel: 173 kb for the 20k panel and 96 kb for the 50k panel.

Haplotype blocks were studied in IllumHD and CP using 809 animals and in AffyHD using 93 animals. The quantity and size of the haplotypes in these panels were compared. Haplotype blocks were reconstructed in CP after imputation. Haplotype phases were obtained using FImpute v.2.2b^17^ for all the panels studied. Thereafter, the criteria used to obtain the haplotype blocks were the same as the criteria used by Gabriel *et al*.^24^, and the criteria were applied to each autosome chromosome using Haploview software^25^. These results were then used to graphically show four classes of haplotype sizes and the locations of these haplotypes across chromosomes for the IllumHD, AffyHD and CP panels. To evaluate possible differences in the haplotype blocks formed by the IllumHD and AffyHD panels, a study with SNPs present in regions with and without haplotype blocks was performed.

## Results

### Imputation

Imputation accuracies calculated by animal are described in Table 1. The COR estimates were higher than the PROP estimates for the same analyses in most cases, but both measurements provided virtually the same information; e.g., the highest accuracy observed with both measurements was from 50kCust3 to IllumHD, and the lowest accuracy was from 20 kCom to AffyHD.

**Table 1:** Accuracy calculated by animal (COR and PROP) and standard deviation (SD) for imputation from low-density to high-density panel. IllumHD – high-density panel from Illumina; AffyHD – high-density panel from Affymetrix; CP – combined panel using IllumHD and AffyHD; 20 kCom – commercial panel containing approximately 20,000 SNPs; 20kCust1, 20kCust2, 20kCust3 - first, second, and third customized panel containing approximately 20,000 SNPs, respectively; 50 kCom - commercial panel containing approximately 50,000 SNPs; 50kCust1, 50kCust2, 50kCust3 - first, second, and third customized panel containing approximately 50,000 SNPs, respectively.

Analysis	Lower panel	Higher panel	SNPs to be imputed	COR (SD)	PROP (SD)
1	IllumHD	CP	371,229	0.84 (0.24)	84.11 (18.39)
2	20 kCom	IllumHD	493,532	0.88 (0.09)	84.96 (5.80)
3	20 kCust1	IllumHD	493,532	0.87 (0.09)	83.89 (5.85)
4	20 kCust2	IllumHD	493,532	0.87 (0.09)	84.34 (5.80)
5	20 kCust3	IllumHD	493,532	0.89 (0.09)	86.22 (5.64)
6	20 kCom	AffyHD	422,682	0.70 (0.18)	68.08 (10.00)
7	20 kCust1	AffyHD	412,300	0.74 (0.20)	73.47 (12.23)
8	20 kCust2	AffyHD	412,300	0.75 (0.20)	74.14 (12.54)
9	20 kCust3	AffyHD	412,300	0.77 (0.20)	75.49 (13.22)
10	20 kCom	CP	864,761	0.84 (0.09)	81.24 (6.22)
11	20 kCust1	CP	864,761	0.83 (0.09)	80.22 (6.05)
12	20 kCust2	CP	864,761	0.83 (0.09)	80.67 (6.10)
13	20 kCust3	CP	864,761	0.84 (0.09)	82.16 (6.26)
14	50 kCom	IllumHD	481,161	0.88 (0.10)	85.94 (6.05)
15	50 kCust1	IllumHD	481,161	0.88 (0.09)	85.99 (5.85)
16	50 kCust2	IllumHD	481,161	0.89 (0.09)	86.96 (5.70)
17	50 kCust3	IllumHD	481,161	0.90 (0.09)	88.21 (5.52)
18	50 kCom	AffyHD	408,880	0.73 (0.19)	72.65 (11.93)
19	50 kCust1	AffyHD	399,929	0.74 (0.20)	73.95 (12.61)
20	50 kCust2	AffyHD	399,929	0.77 (0.21)	75.93 (13.40)
21	50 kCust3	AffyHD	399,929	0.78 (0.21)	76.91 (13.95)
22	50 kCom	CP	852,390	0.84 (0.09)	81.93 (6.31)
23	50 kCust1	CP	852,390	0.84 (0.09)	82.00 (6.33)
24	50 kCust2	CP	852,390	0.85 (0.09)	82.87 (6.43)
25	50 kCust3	CP	852,390	0.86 (0.09)	83.87 (6.55)

The accuracies obtained with the higher-density panel IllumHD using only 70 animals were similar to those obtained using 786 animals, with a difference of 0.01 for COR and one unit for PROP. These results suggest that the accuracies observed for 70 animals, when AffyHD and CP were the high-density panels, are expected for the whole population. In general, the lowest imputation accuracies were obtained for AffyHD, followed by CP and IllumHD. With the exception of analysis 1 (IllumHD to CP), the increased number of SNPs at a lower density increased the imputation accuracy.

IllumHD to CP had high imputation accuracy but also the highest standard deviation observed in this study (Table 1). Even with the much larger number of SNPs to be imputed to CP with the lower-density 20k and 50k panels, they had essentially the same imputation accuracies when compared to IllumHD to CP.

In general, the comparison of commercial panels and customized panels revealed that the third method of customization was best, with increased imputation accuracy for almost all imputations studied. The use of Cust3 resulted in a greater increase in the accuracy of imputations to the AffyHD panel (0.70 to 0.77 for 20k and 0.73 to 0.78 for 50k) when compared to imputations to IllumHD and CP.

Considering the imputations using 20k as the lower-density panel, the first and second methods of customization had imputation accuracies to CP and IllumHD that were lower than those to the 20k commercial panel. However, higher accuracies were observed for these two methods of customizing panels when compared with commercial panels for imputation to AffyHD.

For imputation of 50k to CP and IllumHD, the estimates were similar for 50 kCom, 50kCust1, and 50kCust2. When considering the imputation of 50k to AffyHD, higher accuracies were observed for 50kCust1 and 50kCust2 when compared with 50 kCom.

**Figure 2:**
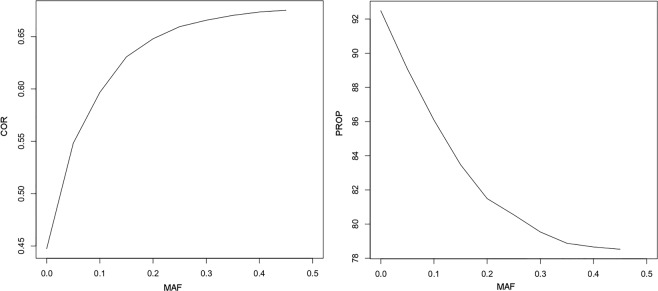
Relation between the accuracy calculated by SNP and minor allele frequency (MAF) for imputation from IllumHD to CP. COR - accuracy measured by allelic correlation and PROP – accuracy measured according to the proportion of genotypes correctly imputed.

The relationship between accuracy calculated by SNP and MAF is demonstrated in Fig. 2 for imputation from IllumHD to CP; however, the imputations from all panels showed similar patterns. The correspondence observed between the accuracy measured by COR and MAF contrasted with that observed for PROP and MAF, i.e., an increasing MAF results in an increase in COR and a decrease in PROP.

The imputation accuracy calculated by SNP differed slightly among chromosomes. For all imputations to IllumHD, the highest accuracies were observed on chromosomes (BTA) 6 and 8, and the lowest accuracies were observed on BTA25. In the case of imputations to AffyHD, the chromosomes with the highest accuracy estimates were BTA5 and BTA6, while BTA19, BTA25, and BTA29 had the lowest accuracies. Since CP represents an aggregate of panels, the chromosomes with high imputation accuracies for IllumHD and AffyHD also revealed high accuracy for CP (BTA5, BTA6, and BTA8). The same pattern was observed for BTA19, BTA25, and BTA29, which again had the lowest accuracies.

### Linkage disequilibrium and haplotype blocks

**Figure 3:**
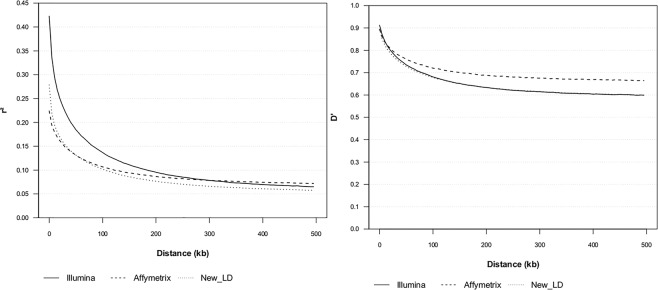
Linkage disequilibrium decay. IllumHD - high-density panel from Illumina; AffyHD - high-density panel from Affymetrix; and New_LD – linkage disequilibrium between the allele pairs that were present in the combined panel and absent from the individual high-density panels.

The LD decay for IllumHD, AffyHD, and New_LD (LD between pairs of alleles that were absent from the individual high-density panels) are shown in Fig. 3. The r^2^ had a different decay value when compared to D′, wherein similar estimates were obtained for AffyHD and IllumHD for short distances between SNPs, and for long distances, the AffyHD panels maintained higher estimates. The New_LD for D′ followed the IllumHD panel. For r^2^, IllumHD revealed higher estimates compared with AffyHD for short distances between SNPs, and the estimate for New_LD was slightly higher when compared with AffyHD for short distances between SNPs. However, with increasing distance, these new combinations showed the lowest estimates. Similar to the sum of the combinations revealed in Fig. 3, the CP (not shown in Figure) had a decay that was intermediate between that of the two high-density panels.

The means of linkage disequilibrium (r^2^) and standard deviations between SNPs that were present at lower density and those that were present only at higher density are described in Table 2. In general, the lowest means for LD were observed for the lower-density panels with AffyHD as the higher-density panel, while the highest LD means were observed for lower-density panels with CP as the higher-density panel. For the 20k and 50k low-density panels, the commercial panel showed the lowest means, while the highest means were observed for the Cust3 panels. The IllumHD panel had 84,529 haplotype blocks, which was greater than that in AffyHD, which had 63,967 haplotype blocks, and fewer than 140,336 blocks were detected in the CP panel. Variability in the mean was detected in different panels, wherein IllumHD was composed of haplotype blocks with a mean of 13.770 ± 21.905 kb and a median haplotype block length of 6.935 kb, AffyHD had a mean of 10.210 ± 15.547 kb and a median of 5.755 kb, and CP had a mean of 10.710 ± 16.914 kb and a median of 5.539 kb.

**Table 2:** Linkage disequilibrium (r^2^) means between SNPs present in low-density panels and between SNPs present only in high-density panels for pairs of SNPs less than 173 kb apart (20k panel) and less than 96 kb apart (50k panel).

Low density	High density		
	IllumHD	AffyHD	CP
20 kCom	0.25 ± 0.29	0.21 ± 0.26	0.28 ± 0.31
20kCust1	0.29 ± 0.32	0.22 ± 0.26	0.31 ± 0.33
20kCust2	0.29 ± 0.31	0.22 ± 0.25	0.30 ± 0.31
20kCust3	0.33 ± 0.33	0.24 ± 0.26	0.33 ± 0.32
50 kCom	0.26 ± 0.32	0.20 ± 0.26	0.28 ± 0.33
50kCust1	0.28 ± 0.32	0.20 ± 0.26	0.29 ± 0.33
50kCust2	0.27 ± 0.31	0.21 ± 0.25	0.29 ± 0.31
50kCust3	0.30 ± 0.32	0.22 ± 0.26	0.30 ± 0.32

**Figure 4:**
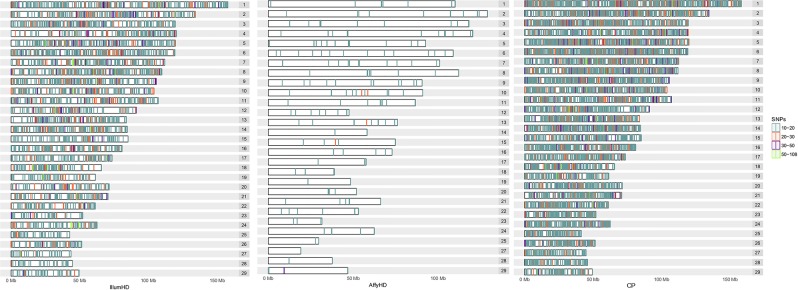
Haplotype blocks composed of more than 10 SNPs distributed within chromosomes. IllumHD - high-density panel from Illumina; AffyHD - high-density panel from Affymetrix; and CP – a combined panel using IllumHD and AffyHD.

Most haplotype blocks of these three panels were composed of fewer than 10 SNPs, with only 3882, 193, and 8462 haplotype blocks composed of at least 10 SNPs detected in IllumHD, AffyHD and CP, respectively (Fig. 4). With the IllumHD panel, 27 haplotype blocks were identified with at least 50 SNPs, and the largest haplotype block contained 95 SNPs with a length of 326.5 kb. For this panel, BTA7 and BTA18 showed more of these larger haplotypes, with three large haplotype blocks. For AffyHD, no haplotype blocks containing more than 50 SNPs were detected. The CP panel contained 38 haplotype blocks with more than 50 SNPs, wherein BTA2, BTA5, BTA7, BTA12, and BTA18 each contained three of these large haplotype blocks. For the CP panel, the largest haplotype contained 108 SNPs with a length of 310.5 kb.

The SNPs present in regions with and without haplotype blocks were evaluated, with an aim to investigate the differences in the number of haplotype blocks observed when using the IllumHD and AffyHD panels. There were 149,923 SNPs (29.45%) in the regions not covered by haplotype blocks for IllumHD and 233,631 SNPs (54.61%) for AffyHD. Additionally, the MAF were calculated, in both panels, for SNPs located in the regions for which large haplotypes were detected when using IllumHD panel, and not detected when using AffyHD panel, to investigate the influence of MAF on haplotype block identification. The first regions studied were the sites where haplotype blocks with more than 50 SNPs were identified when using IllumHD. In these regions, the number of SNPs from each panel and the respective proportion were calculated for different MAF classes. The same was performed for regions for which haplotype blocks with 30 to 50 SNPs were detected when IllumHD was used. Finally, the number of SNPs and their proportion for each MAF class were calculated for regions in which both panels identified haplotype blocks. A higher proportion of SNPs with MAF lower than 0.05 was observed for AffyHD panel (0.13 and 0.17) comparing to IllumHD panel (0.06 and 0.08) in regions which large haplotypes were only detected when using IllumHD panel (Table 3). The same proportion of SNPs with low MAF for both panels (0.10) was identified in regions with common haplotype (Table 3).

**Table 3:** Number of SNPs (N) and proportion of SNPs (P) according to minor allele frequency (MAF) classes in different regions covered by haplotype blocks calculated for the high-density panel from Illumina (IllumHD) and high-density panel from Affymetrix (AffyHD). IllumHD haplotype (more than 50 SNPs)—regions covered by haplotype blocks with more than 50 SNPs when using the IllumHD panel; IllumHD haplotype (30 to 50 SNPs)—regions covered by haplotype blocks with 30 to 50 SNPs when using the IllumHD panel; haplotype in common (IllumHD and AffyHD)—regions for which both panels identified haplotype blocks.

Regions	Description	Panel	MAF Classes						
			0 −| 0.01	0.01 −| 0.05	0.05 −| 0.1	0.1 −| 0.2	0.2 −| 0.3	0.3 −| 0.4	0.4 −| 0.5
IllumHD haplotype (more than 50 SNPs)	N	IllumHD	35	64	156	638	329	167	364
		AffyHD	12	69	70	164	106	86	111
	P	IllumHD	0.02	0.04	0.09	0.36	0.18	0.11	0.20
		AffyHD	0.02	0.11	0.11	0.27	0.17	0.14	0.18
IllumHD haplotype (30 to 50 SNPs)	N	IllumHD	125	470	864	2199	1380	1012	1113
		AffyHD	84	435	335	793	478	499	475
	P	IllumHD	0.02	0.06	0.12	0.31	0.19	0.14	0.16
		AffyHD	0.03	0.14	0.11	0.26	0.15	0.16	0.15
Haplotype in common (IllumHD and AffyHD)	N	IllumHD	1701	7855	10287	21752	18460	16761	15316
		AffyHD	1397	6224	5830	17632	14916	17764	15823
	P	IllumHD	0.02	0.08	0.11	0.24	0.20	0.18	0.17
		AffyHD	0.02	0.08	0.07	0.22	0.19	0.22	0.20

## Discussion

### Imputation

The structure of this population was previously described in another study, which reported that the genomic diversity in this population is not high enough to differentiate among families^20^. Although there were relatively few animals in the reference population, which resulted in lower imputation accuracies when compared to those in other studies^8^, the presence of the sires helped the software to reconstruct haplotypes. Khatkar *et al*.^13^ studied dairy cattle and observed that imputation with sires in the reference population resulted in a slightly lower allelic error rate when compared to imputation for animals without sires. The same results were observed in the present study, wherein the means of COR calculated for animals with sires in the reference population showed values slightly higher than the means of COR calculated for animals without a sire in reference population for almost all the imputations studied. The largest difference in these means was 0.06. Lower values observed for the PROP measurements when compared with the COR measurements were also reported by Carvalheiro *et al*.^8^; according to the authors, the higher penalty given to one incorrectly imputed allele in the first measurement can lead to the observed result.

The highest accuracy estimate was obtained in analysis 17 (50kCust3-IllumHD), which was likely due to the density summed with the high LD observed between SNPs that were present at a low density and SNPs that were present only at a high density. Although the LD (Table 2) estimates for 20kCust1-CP, 20kCust3-IllumHD, and 20kCust3–CP were higher than that estimated for 50kCust3-IllumHD, the lower number of SNPs to be imputed using the 50k density panels (versus the 20k panel) allowed the software to improve the haplotype inference, which in turn reduced the imputation error rates. According to Pei *et al*.^18^, several factors can influence imputation accuracy, but LD plays a central role in the methods evaluated by those authors. The same factors (linkage disequilibrium and SNP density) may have contributed to the lowest imputation accuracy being estimated by using the 20 kCom-AffyHD (analysis 6), wherein, among the lowest estimates of LD shown in Table 2, 20 kCom is the lowest SNP density panel.

The selection of SNPs to compose the panels differed between IllumHD and AffyHD, and this may have resulted in difficulties in obtaining a standard to form the haplotypes, leading to differences in imputation accuracy across the genome. Therefore, animals with a relatively lower relationships with animals in the reference population may present greater difficulty in regard to the identification of haplotypes and consequently result in lower imputation accuracy. This is reflected by the high standard deviation observed for the IllumHD-CP imputation. The similar estimated accuracies for imputation from the panels with 20k and 50k densities to CP compared with imputation from IllumHD to CP can be explained by LD decay. The New_LD (Fig. 3), even over short distances between SNPs, revealed LD estimates lower than those for IllumHD, which indicates that the SNPs from IllumHD have low LD with SNPs from AffyHD, and these are the SNPs that need to be imputed in IllumHD-CP (Analysis 1). In the 20k-CP and 50k-CP imputations, there are two types of SNPs that need to be imputed. The first type is composed of the SNPs that are present in CP and absent in IllumHD, which need to be imputed in all three cases: IllumHD-CP, 20k-CP and 50k-CP. This type of SNP seems more difficult to impute, based on the New_LD pattern (Fig. 3). The second type is composed of SNPs that are present in CP and IllumHD but absent in the 20k and 50k panels. This second type of SNP seems to be more easily imputed because Illumina LD measured by r^2^ (Fig. 3) is relatively high. Therefore, the second type of SNPs may help to increase the mean accuracies of 20k-CP and 50k-CP imputations, leading to a value similar to that observed for IllumHD-CP.

Similar accuracies were calculated using either 70 or 786 animals, likely due to the structure of the population, wherein values obtained for animals with close relationships are similar. SNP and LD factors can explain the high imputation accuracy estimates for IllumHD, wherein LD estimates were lower than those for CP; however, fewer SNPs need to be imputed. The third method of panel customization seems to be the most appropriate due to the inclusion of two factors that affect the accuracy of imputation: LD and MAF. The selection of SNPs with a higher LD than other SNPs in the window can contribute to haplotype reconstruction.

Comparing imputation accuracies among the commercial and customized panels revealed that the increase in accuracies was greatest when the third method of panel customization for imputation to AffyHD was used (Table 1). This panel excluded SNPs with a low MAF, consequently, these SNPs needed to be imputed. According to Pei *et al*.^18^, the influence of MAF can be high in low-LD regions, wherein in these regions, markers with a low MAF likely revealed locally high levels of LD with nearby markers, although the region as a whole has low LD. Because the AffyHD panel revealed a lower mean LD (Table 2) and contained more regions with low LD, the SNPs with a low MAF that needed to be imputed with 20kCust3 and 50kCust3 could enhance the improvement associated with the use of this customized panel for AffyHD. Due to the influences of MAF on imputation accuracy, a customization of a panel using SNPs in common between IllumHD and AffyHD and considering the LD between these SNPs and SNPs present in the high-density panels could improve the accuracies.

The study of imputation accuracy versus MAF (Fig. 2) indicated different patterns for COR and PROP. These results were also observed by Ma *et al*.^26^, and those authors noted that the PROP measurement does not consider correct imputation by chance, thus favoring SNPs with a low MAF. The authors reported that, for these reasons, the correlation can better capture the imputation accuracy, particularly for SNPs with a low MAF.

Piccoli *et al*.^27^ suggested that chromosome length is related to imputation accuracy. In the present study, performance differed among chromosomes for both high-density panels and the combined panel. This suggests that not only the size but also the haplotype structure can influence the performance of imputation across chromosomes. When the imputation was to IllumHD, the shortest chromosome among the autosomes, BTA25, revealed the lowest estimates of imputation accuracy. However, although BTA6 and BTA8 were not the longest chromosomes, they showed the highest estimated accuracy. These are among the five chromosomes with the highest number of haplotypes containing more than 10 SNPs for IllumHD. They are also the top two chromosomes when considering the number of haplotypes divided by the size of the chromosome (density; Fig. 4). In addition, these haplotypes are concentrated in the middle of the chromosome, and according to Sun *et al*.^28^, it is relatively difficult to impute SNPs present at the beginning and the end of a chromosome. The number of haplotypes also seems to influence the imputation for AffyHD, wherein the highest estimates for imputation accuracy were observed for the chromosomes with the greatest number of haplotypes containing more than 10 SNPs (BTA5 and BTA6; Fig. 4). The CP, as an aggregation of two high-density panels, showed the highest imputation accuracies for chromosomes BTA5, BTA6 and BTA8, which represent the combination of chromosomes with the highest accuracy observed for the IllumHD (BTA6 and BTA8) and AffyHD (BTA5 and BTA6) panels.

### Linkage disequilibrium and haplotype blocks

Higher LD estimates were observed for D′ when compared with r^2^. According to Espigolan *et al*.^29^, D′ can overestimate LD. The authors also reported that one disadvantage of D′ is that it is strongly overestimated when small samples and SNPs with a low MAF are used. This can explain the highest estimates of D′ being observed for the AffyHD panel. Although it contained fewer SNPs with a low MAF, the sample used to calculate LD was smaller than that used to calculate LD for the other two panels.

In general, the r^2^ estimates for IllumHD were slightly lower than those reported by O’Brien *et al*.^30^ for Nelore cattle. These differences may have occurred because the LD means were calculated for every 1 kb, differing from the present study, in which they were calculated for every 5 kb. Even considering imprecise estimation due to the small sample size, the AffyHD r^2^ values at short distances were considerably lower than those for the IllumHD panel. According to Van Binsbergen *et al*.^31^, large differences in MAF for a pair of SNPs can result in low LD estimates, even when the distance between SNPs is small. For the IllumHD panel, 53% of the SNP pairs revealed a difference in MAF of greater than 0.1, while the estimate for AffyHD was 61%, which may explain the low LD estimates for the AffyHD panel over short distances between SNPs.

The AffyHD panel contained the lowest number of haplotype blocks, and the length of the blocks was small. The MAF for SNPs in the two high-density panels may explain the differences in the number of haplotype blocks. In the AffyHD panel, a low MAF was observed for SNPs located in regions for which haplotype blocks were detected when using IllumHD but not detected when using AffyHD. When evaluating SNPs located in the regions where haplotypes with more than 50 SNPs were detected using IllumHD, a higher proportion of SNPs with a MAF lower than 0.05 was observed for AffyHD (13%) when compared to IllumHD (6%). The same was observed when evaluating the regions for which there were haplotypes with 30 to 50 SNPs for IllumHD. In these regions, only 8% of the SNPs from IllumHD showed a MAF lower than 0.05, while for AffyHD, the percentage was 17% of SNPs. However, when studying regions for which haplotypes were observed in common for IllumHD and AffyHD, the percentage of SNPs with a MAF lower than 0.05 was 10% for both panels. These descriptions suggest the influences of the MAF in the differences of reconstructions of haplotypes when using IllumHD rather than AffyHD.

The combination of the two high-density panels seems to contribute to the increase in the number of haplotype blocks, which may be important for imputation in *Bos taurus indicus*, even considering that the average size was smaller for CP than for IllumHD. The presence of more haplotype blocks may increase the accuracy of imputation for these regions, as observed for IllumHD-CP imputation, wherein 35% of the SNPs located in the haplotype blocks composed of at least 10 SNPs showed accuracy higher than 0.70, while this high accuracy was observed for only 21% of the SNPs located in other regions. The median haplotype block size for CP indicated that most haplotype blocks were smaller than the average, but the increase in the number of long haplotypes used in this panel in comparison to IllumHD and the increase in the number of haplotypes observed on small chromosomes (BTA23 to BTA29; Fig. 4) can contribute to studies that utilize haplotype blocks. According to Cuyabano *et al*.^32^, the advantage of using haplotype blocks instead of using individual SNP information in genomic selection is that each haplotype may have higher LD with a causal mutation than any individual SNP.

## Conclusion

In general, the use of SNPs in a combined panel is recommended due to the increased density and number of haplotype blocks. Considering common SNPs between IllumHD and AffyHD for the customization of a low-density panel increases the imputation accuracy for IllumHD, AffyHD and CP.

## Data Availability

The datasets generated during and/or analysed during the current study are available in the Figshare repository, https://figshare.com/articles/Evaluation_of_imputation_accuracy_using_the_combination_of_two_high_density_panels_in_Nelore_beef_cattle/7140347. The files available contain genotype information following quality control, performed as described in the “Methods” section. The files are in Plink format. Each dataset contains three files with the following extensions: *.bed, *.bim, *.fam. The names of files correspond to the respective panels used. The genotypes are available in the figshare repository, and the description and accession numbers are listed in File S1.
